# UMI-tools: modeling sequencing errors in Unique Molecular Identifiers to improve quantification accuracy

**DOI:** 10.1101/gr.209601.116

**Published:** 2017-03

**Authors:** Tom Smith, Andreas Heger, Ian Sudbery

**Affiliations:** 1 Computational Genomics Analysis and Training Programme, MRC WIMM Centre for Computational Biology, University of Oxford, Oxford OX3 9DS, United Kingdom;; 2 Department of Molecular Biology and Biotechnology, University of Sheffield, Sheffield S10 2TN, United Kingdom

## Abstract

Unique Molecular Identifiers (UMIs) are random oligonucleotide barcodes that are increasingly used in high-throughput sequencing experiments. Through a UMI, identical copies arising from distinct molecules can be distinguished from those arising through PCR amplification of the same molecule. However, bioinformatic methods to leverage the information from UMIs have yet to be formalized. In particular, sequencing errors in the UMI sequence are often ignored or else resolved in an ad hoc manner. We show that errors in the UMI sequence are common and introduce network-based methods to account for these errors when identifying PCR duplicates. Using these methods, we demonstrate improved quantification accuracy both under simulated conditions and real iCLIP and single-cell RNA-seq data sets. Reproducibility between iCLIP replicates and single-cell RNA-seq clustering are both improved using our proposed network-based method, demonstrating the value of properly accounting for errors in UMIs. These methods are implemented in the open source UMI-tools software package.

High-throughput sequencing technologies yield vast numbers of short sequences (reads) from a pool of DNA fragments. Over the last ten years, a wide variety of sequencing applications have been developed that estimate the abundance of a particular DNA fragment by the number of reads obtained in a sequencing experiment (read counting) and then compare these abundances across biological conditions. Perhaps the most widely used read counting approach is RNA-seq, which seeks to compare the number of copies of each transcript in different cell types or conditions. Prior to sequencing, a PCR amplification step is normally performed to ensure sufficient DNA for sequencing and/or enrichment for fragments with successful adapter ligation. Biases in the PCR amplification step lead to particular sequences becoming overrepresented in the final library (Aird et al. 2011). In order to prevent this bias propagating to the quantification estimates, it is common to remove reads or read pairs with the same alignment coordinates, because they are assumed to arise through PCR amplification of the same molecule (Sims et al. 2014). This is appropriate where sequencing depth is low and thus the probability of two independent fragments having the same genomic coordinates are low, as with paired-end whole-genome DNA-seq from a large genome. However, the probability of generating independent fragments mapping to the same genomic coordinates increases as the distribution of the alignment coordinates deviates from a random sampling across the genome and/or the sequencing depth increases. For example, in RNA-seq, highly expressed transcripts are more likely to generate multiple fragments with exactly the same genomic coordinates. The problem of PCR duplicates is more acute when greater numbers of PCR cycles are required to increase the library concentration, as in single-cell RNA-seq, or when the alignment coordinates are limited to a few distinct loci, as in individual-nucleotide resolution Cross-Linking and ImmunoPrecipitation (iCLIP). Random barcodes were initially proposed as a method to count the number of mRNA molecules in a sample (Hug and Schuler 2003), and have since been used to explicitly label PCR duplicates (McCloskey et al. 2007). More recently, random barcodes, referred to as Unique Molecular Identifiers (UMIs), have been used to confidently identify PCR duplicates in high-throughput sequencing experiments (König et al. 2010; Kivioja et al. 2012; Islam et al. 2014). By incorporating a UMI into the same location in each fragment during library preparation, but prior to PCR amplification, it is possible to accurately identify true PCR duplicates because they have both identical alignment coordinates and identical UMI sequences (Fig. 1A). In addition to their use in single-cell RNA-seq and iCLIP (König et al. 2010), UMIs may be applied to almost any sequencing method in which confident identification of PCR duplicates by alignment coordinates alone is not possible and/or an accurate quantification is required, including ChIP-exo (He et al. 2015), DNA-seq karyotyping (Karlsson et al. 2015), detection of rare mutations (Schmitt et al. 2012), and antibody repertoire sequencing (Vollmers et al. 2013).

**Figure 1.**
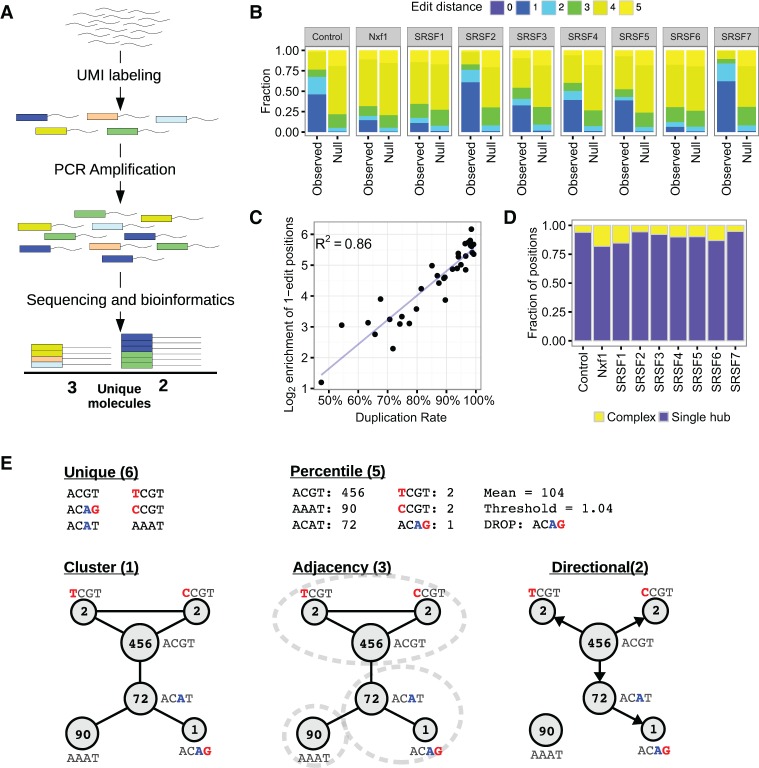
Modeling errors in UMIs. (*A*) Schematic representation of how UMIs are used to count unique molecules. Fragmented DNA is labeled with a random UMI sequence (short oligonucleotide; represented as colored blocks). Following PCR amplification, sequencing, and bioinformatics steps, the sequence read alignment coordinates and UMI sequences are used to identify sequence reads originating from the same initial DNA fragment (PCR duplicates) and so count the unique molecules. (*B*) Average edit distances (rounded to integers) between UMIs with the same alignment coordinates. Genomic positions with a single UMI are not shown. (Null) Null expectation from random sampling of UMIs, taking into account the genome-wide distribution of UMIs. (*C*) Correlation between duplication rate and enrichment of positions with an average edit distance of 1 for iCLIP data. (*D*) Topologies of networks formed by joining reads with the same genomic coordinates and UMIs a single edit distance apart. (Single hub) One node connected to all other nodes; (complex) no node connected to all other nodes. (*E*) Methods for estimating unique molecules from UMI sequences and counts at a single locus. Where the method uses the UMI counts, these are shown. Red bases are inferred to be sequencing errors, and blue bases are inferred to be PCR errors. The inferred number of unique molecules for each method is shown in parentheses.

Accurate quantification with UMIs is predicated on a one-to-one relationship between the number of unique UMI barcodes at a given genomic locus and the number of unique fragments that have been sequenced. However, errors within the UMI sequence, including nucleotide substitutions during PCR and nucleotide miscalling and insertions or deletions (indels) during sequencing, create additional artifactual UMIs. Nucleotide miscalling and substitution errors affect only the UMI sequence itself and do not affect the alignment coordinates. Hence, these errors will inflate the estimation of the number of unique molecules at a particular genomic coordinate. These errors can be identified by examining all UMIs at a single genomic coordinate. On the other hand, UMI indels will affect the alignment position also, leading to the assignment of reads to incorrect genome coordinates. Identification of such events requires the examination of sets of UMIs at neighboring coordinates. Recombination events, also called “PCR jumping,” create chimeric sequences that may change either the UMI sequence and/or alignment. Miscalling during sequencing is by far the most prevalent error, occurring one to two orders of magnitude more frequently than indels for Illumina sequencing (Marinier et al. 2015; Schirmer et al. 2015, 2016). Recombination is common when sequencing amplicons, but much rarer with the shotgun sequencing approaches in which UMIs are utilized (Schloss et al. 2011; Waugh et al. 2015). We therefore focus here on improving quantification via UMIs by considering nucleotide miscalling and substitution errors within pools of UMIs from the same genomic coordinate. Herein, we will refer to these errors as UMI errors.

UMI errors have been considered in previous analyses (Islam et al. 2014; Bose et al. 2015; Macosko et al. 2015; Yaari and Kleinstein 2015). However, their impact on quantification accuracy has not previously been demonstrated, and there is no consistency in the approach taken to resolve these errors. For example, Islam et al. (2014) removed all UMIs where the counts were >1% of the mean counts of all other nonzero UMIs at the genomic locus, whereas Bose et al. (2015) merged together all UMIs within a Hamming distance of two or less, with little explanation as to how this was achieved. We therefore set out to demonstrate the need to account for UMI errors, to compare different methods for resolving UMI errors and to formalize an approach for removing PCR duplicates with UMIs.

## Results

We reasoned that UMI errors create groups of similar UMIs at a given genomic locus. To confirm this, we calculated the average number of bases different (edit distance) between UMIs at a given genomic locus and compared the distribution of average edit distances to a null distribution generated by randomly sampling (Methods). Using iCLIP data (Müller-McNicoll et al. 2016), we confirmed that the UMIs are more similar to one another than expected according to the null, strongly suggesting sequencing and/or PCR errors are generating artifactual UMIs (Methods; Fig. 1B; for other data sets, see Supplemental Fig. S1). Furthermore, the enrichment of low edit distances is well correlated with the degree of PCR duplication (Fig. 1C). Overall, we detected a 25-fold enrichment for positions with an average edit distance of 1, compared to our null expectation. In contrast, when we compared the UMI sequences at adjacent positions, we detected an 1.1-fold (± standard deviation of 0.1) (Methods) enrichment for UMIs, which may have originated from a single nucleotide deletion, suggesting UMI indels are much less prevalent than UMI errors, as expected. We then constructed networks between UMIs at the same genomic locus, where nodes represent UMIs and edges connect UMI separated by a single nucleotide difference. Although most of the networks contained just a single node, we observed that 3%–36% of networks contained two or more nodes, of which 4%–20% did not contain a single central node, and thus could not be naïvely resolved (Fig. 1D). This indicates that the majority of networks are likely to originate from a single unique molecule prior to PCR amplification, but a minority of networks may originate from a combination of errors during PCR and sequencing or may originate from multiple unique molecules, which by chance have similar UMIs.

### Methods to identify unique molecules

Many previous studies assume each UMI at a given genomic locus represents a different unique molecule (Shiroguchi et al. 2012; Soumillon et al. 2014; Collins et al. 2015). We refer to this method as *unique*. Islam et al. (2014) previously identified the issue of sequencing errors and proposed removing UMIs whose counts fall below a threshold of 1% of the mean of all nonzero UMIs at the locus, a method we refer to as *percentile*.

We have developed three methods to identify the number of unique molecules at a given locus by resolving UMI networks formed by linking UMIs separated by a single edit distance (Fig. 1E). In all cases, the aim is to reduce the network down to a representative UMI(s) that accounts for the network; identifying the exact sequence of the original UMI(s) is not important for the purposes of quantification. The simplest method we examined was to merge all UMIs within the network, retaining only the UMI with the highest counts. For this method, the number of networks formed at a given locus is equivalent to the estimated number of unique molecules. This is similar to the method used by Bose et al. (2015) where UMIs with an edit distance of 2 or less were considered to originate from an identical molecule. We refer to this method as *cluster*. This method is expected to underestimate the number of unique molecules, especially for complex networks. We therefore developed the *adjacency* method, which attempts to correctly resolve the complex networks by using the node counts. The most abundant node and all nodes connected to it are removed from the network. If this does not account for all the nodes in the network, the next most abundant node and its neighbors are also removed. This is repeated until all nodes in the network are accounted for. In the method, the total number of steps to resolve the network(s) formed at a given locus is equivalent to the number of estimated unique molecules. This method allows a complex network to originate from more than one UMI, although UMIs with an edit distance of two will always be removed in separate steps. The excess of UMIs pairs with an edit distance of two observed in the iCLIP data sets indicate that some of these UMIs are artifactual. Reasoning that counts for UMIs generated by a single sequencing error should be higher than those generated by two errors and UMIs resulting from errors during the PCR amplification stage should have higher counts than UMIs resulting from sequencing errors, we developed a final method, *directional*. We generated networks from the UMIs at a single locus, in which directional edges connect nodes a single edit distance apart when *n*_a_ ≥ 2*n*_b_ − 1, where *n*_a_ and *n*_b_ are the counts of node a and node b. The entire directional network is then considered to have originated from the node with the highest counts. The ratio between the final counts for the true UMI and the erroneous UMI generated from a PCR error is dependent upon which PCR cycle the error occurs and the relative amplification biases for the two UMIs, but should rarely be less than twofold. The −1 component was included to account for strings of UMIs with low counts, each separated by a single edit distance for which the 2*n* threshold alone is too conservative. This method allows UMIs separated by edit distances greater than one to be merged so long as the intermediate UMI is also observed, and with each sequential base change from the most abundant UMI, the count decreases. For this method, the number of directional networks formed is equivalent to the estimated number of unique molecules.

### Comparing methods with simulated data

**Figure 2.**
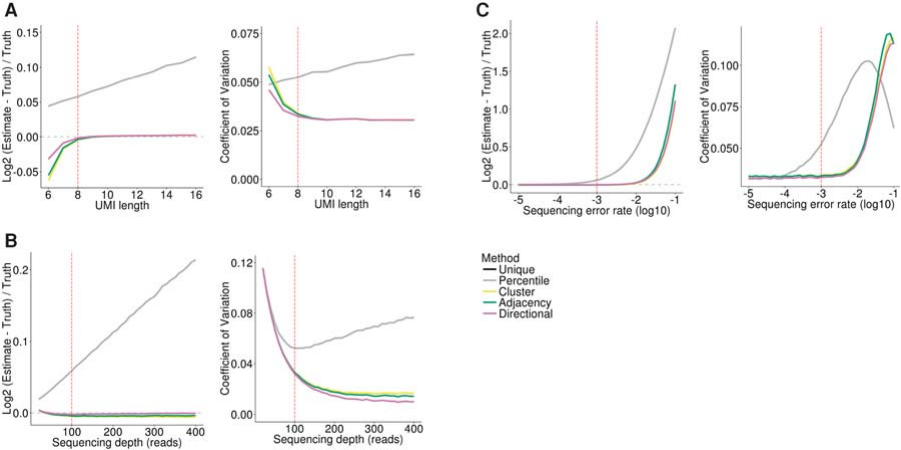
Comparison of methods with simulated data. In each panel, all but one of the simulation parameters are held constant, with the remaining parameter varied as shown on the *x*-axis. (*A*) UMI length. (*B*) Sequencing depth. (*C*) Sequencing error rate. The *left* plots show the accuracy of quantification, presented as the log_2_-transformed normalized difference between the estimate and ground truth. The *right* plots show the coefficient of variation (standard deviation/mean). The dashed red line represents the value used for this parameter in all other simulations. The dashed gray line represents perfect accuracy. The *unique* and *percentile* methods give identical results with the parameters shown here and are hence overplotted.

To compare the accuracy of the proposed methods, we simulated the process of UMI amplification and sequencing for UMIs at a single locus and varied the simulation parameters (Methods). To examine the accuracy of the five methods, we computed two metrics: the log_2_-fold difference between the estimate and ground truth log_2_[(estimate − truth)/truth] and the coefficient of variation (CV) (standard deviation/mean) across 10,000 iterations. Increasing UMI length or sequencing depth results in a linear increase in the degree of overestimation for *unique* and *percentile* (Fig. 2A,B), since increasing either parameter linearly increases the total amount of UMI sequence that may harbor errors. In contrast, the estimates from the network-based methods remain relatively stable, with *directional* showing the highest accuracy and lowest variance. We also simulated the effect of including a very long UMI (up to 50 bp) as there may be occasions where it is preferable to concatenate a UMI with another barcode, such as a sample barcode or cell barcode in single-cell RNA-seq, leading to longer barcodes. We noted that the network-based methods showed reduced accuracy for very long barcodes (Supplemental Fig. S2A). Investigating further, we found this was correlated with an increase in UMIs with two errors in which the single error intermediate was not observed, as detected by counting the number of networks that did not contain any of the initial UMIs prior to PCR and sequencing (Supplemental Fig. S2B). In order to resolve this inherent problem with very long UMIs, we modified the network-based methods so that edges joined nodes with an edit distance less than or equal to 2. This considerably decreased the number of networks without any initial UMIs and improved the accuracy of the network-based methods for very long UMI sequences (Supplemental Fig. S2A,B).

Increased sequencing error rate leads to an exponential overestimation for *unique* and *percentile* (Fig. 2C), with a 1.3-fold overestimation observed with an error rate of 0.01, compared to <1.05-fold for the network based methods. Increasing the rate of errors during the PCR step had a similar impact (Supplemental Fig. S2C). However, this was only observed when the rate of DNA polymerase errors was simulated as >0.001, considerably higher than reported error rates for even nonrecombinant *Taq* DNA polymerase (Rittié and Perbal 2008; Whalen et al. 2016), confirming sequencing errors are likely to be the primary source of UMI errors. Increasing the number of PCR cycles or modifying the amplification bias had little impact on the relative accuracy of the methods (Supplemental Fig. S2D,E). Increasing the number of initial UMIs reduced the accuracy of the network-based methods; however, even with 100 initial 8-bp UMIs at a single locus, the network methods remained the most accurate (Supplemental Fig. S2F).

Although the network methods performed very similarly, *directional* consistently yielded more accurate and less variable estimates. For example, when the sequencing depth was increased to 400 reads, the average estimates were 19.92, 19.94, and 19.99 (truth = 20), respectively, for *cluster*, *adjacency*, and *directional* methods, and the CVs were 0.0167, 0.0144, and 0.0099. We observed no difference between *percentile* and *unique* under most conditions tested. Increasing the number of reads sequenced per initial UMI, we were able to see an improvement in accuracy for *percentile* relative to *unique* when sequencing error rates are between 1 × 10^−3^ and 1 × 10^−5^; however, even under this specific parameterization, the network-based methods are more accurate (Supplemental Fig. S2G).

In summary, under simulation conditions, the *directional* method outperforms all other methods; however, *adjacency* and *cluster* perform equally well under simulation conditions that are expected to reflect a well-designed and well-executed experiment.

### Implementation

To implement our methods within the framework of removing PCR duplicates from BAM alignment files, we developed a command line tool set, UMI-tools, with two commands, *extract* and *dedup*. *extract* takes the UMI from the read sequence contained in a FASTQ read sequence and appends it to the read identifier so it is retained in the downstream alignment. *extract* expects the UMIs to be contained at the same location in each read. Where this is not the case, e.g., with sequencing techniques such as inDrop-seq (Klein et al. 2015), the user will need to extract the UMI sequence from the read sequence and append it to the read identifier. *dedup* takes an alignment BAM file, identifies reads with the same genomic coordinates as potential PCR duplicates, and removes PCR duplicates using the UMI sequence according to the method chosen. Time requirements for running *dedup* depend on number of input reads, length of UMI, and level of duplication. Memory requirements depend on the number of output reads. On a desktop with a Xeon E3-1246 CPU, it takes ∼220 sec and ∼100 MB RAM to process a 32-million-read single-end input file with 5-bp UMIs to ∼700,000 unique alignments. Inputs with longer UMIs may take significantly longer.

### Comparing methods with iCLIP data

**Figure 3.**
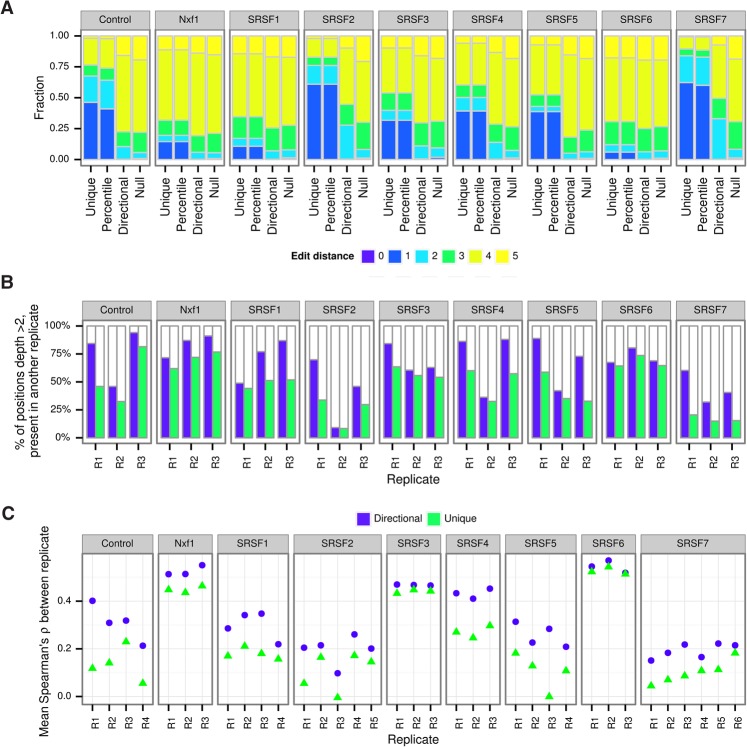
UMI-tools improves reproducibility between iCLIP replicates. (*A*) Average edit distances between UMIs with the same alignment coordinates. Genomic positions with a single UMI are not shown. (Null) Null expectation from random sampling of UMIs, taking into account the genome-wide distribution of UMIs. Only the first replicate of the data set is shown for each pull down. (*B*) iCLIP reproducibility as represented by the percentage of positions with more than two tags also cross-linked in at least one of two other replicates. (*C*) Spearman's rank correlation between the numbers of significant tags in each exon.

We next sought to examine the effect of these methods on real data, starting with the previously mentioned iCLIP data, which includes 3–6 replicates for nine proteins (Müller-McNicoll et al. 2016). For replicate 1, the distribution of the average edit distance between UMIs present at each genomic locus showed enrichment for single-edit distance relative to a null distribution from random sampling, taking into account the genome-wide distribution of UMIs (Fig. 3A). For all samples, application of the *directional* method resulted in an edit distance distribution resembling the null, whereas using the *percentile* method made little or no difference. The same was also true of other replicates of this data set or other data sets (Supplemental Fig. S2). In some cases, a residual enrichment of positions with an average edit distance of 2 was observed, but this was also reduced in most cases.

We reasoned that if the *directional* method removed PCR duplicates more accurately, the reproducibility between replicates should be improved. To test this, we turned to a previously defined measure of iCLIP reproducibility (König et al. 2010). Briefly, we identified in each sample the bases with two or more tags mapping at that position and asked what percentage had a tag present in one or more other replicates for that pull down. We limited the analysis to the first three replicates for each protein. In each case, after de-duplication with the *directional* method, bases with two or more tags were more reproducible (Fig. 3B), with the difference being very large in some cases (e.g., 21% vs. 59% of bases reproducible for SRSF7 replicate 1). In contrast, the *percentile* method was little different from *unique* (Supplemental Fig. S3).

In order to measure reproducibility of their data, Müller-McNicoll et al. (2016) measured the Spearman's rank correlation between the numbers of significant tags in each exon across the genome. We repeated this calculation with data processed, using either the *unique* or *directional* method, and compared the average Spearman's correlation between each sample and other replicates of the same pull down. In all cases, we see an improvement in the correlation between replicates of the same pull down when data are processed using the *directional* method (Fig. 3C). As expected, the degree of improvement for a particular sample was correlated with the enrichment of positions with an average edit distance of 1 (*R*^2^= 0.4) (Supplemental Fig. S3). Thus our method substantially improves the reproducibility of replicates in this iCLIP experiment.

### Comparing methods with single-cell RNA-seq data

**Figure 4.**
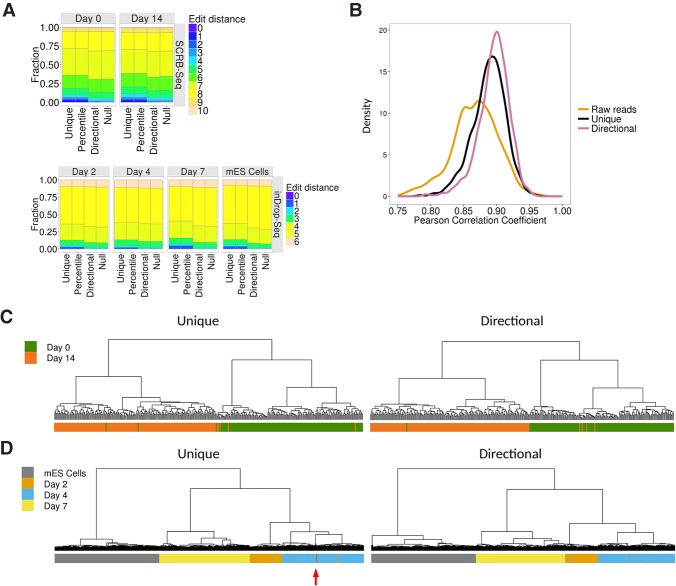
UMI-tools improves accuracy and clustering in single-cell RNA-seq. (*A*) Average edit distances between UMIs with the same alignment coordinates following removal of PCR duplicates using the methods indicated on the *x*-axis. Genomic positions with a single UMI are not shown. (Null) Null expectation from random sampling of UMIs, taking into account the genome-wide distribution of UMIs; (*top*) SCRB-seq; (*bottom*) inDrop-seq. (*B*) Distribution of Pearson correlation coefficients between log ERCC concentration and log counts for raw reads (UMIs ignored) and *unique* and *directional* methods. (*C*,*D*) Hierarchical clustering based on the gene expression estimates obtained using *unique* and *directional*. Color bars represent differentiation stage. (*C*) SCRB-seq. (*D*) inDrop-DSeq. The red arrow indicates mES cells clustering with Day 4 cells.

To further demonstrate the utility of our network-based method, we applied it to two differentiation single-cell RNA-seq data sets: the first reported use of UMIs in a single-cell RNA-seq experiment seeking to describe a developmental pathway (Soumillon et al. 2014), referred to here as SCRB-seq, and a recently reported single-cell RNA-seq utilizing droplet-barcoding (Klein et al. 2015), referred to here as inDrop-seq. As before, network-based methods show a marked improvement in the distribution of edit distances over the *percentile* method and the *unique* method (Fig. 4A). Improvements are generally less pronounced than observed with the iCLIP data, likely due to a lower maximum read depth in single-cell RNA-seq. To demonstrate that this improvement in the edit distance led to an improved accuracy in transcript abundance estimates, we used the ERCC spike-ins. The naïve use of UMIs to identify PCR duplicates with the *unique* method improved the per-cell correlation between ERCC concentration and counts, compared to quantification without considering PCR duplicates (median coefficients were 0.86 and 0.89, respectively). As expected, the correlation was further improved using the *directional* method (median coefficient = 0.91) (Fig. 4B).

We applied hierarchical clustering to the SCRB-seq gene expression data using the *unique* method and observed the Day 0 and Day 14 cells separated relatively well (Fig. 4C). However, seven cells clustered with cells of the wrong time point, reflecting either a failure to commit to differentiation or misclassification event due to noise in the expression estimates. With the *directional* method, this was reduced to five cells, suggesting that failure to account for UMI errors can lead to misclassification in single-cell RNA-seq. Applying hierarchical clustering to the inDrop-seq gene expression estimates, we observed that 44/2717 (1.6%) of cells clustered with cells from another time point when using the *unique* method. Biological variation in the progression of differentiation may explain Day 2, Day 4, and Day 7 misclassification events. However, 19/44 events involved undifferentiated mES cells, suggesting these misclassification events were the result of low-accuracy quantification estimates (Fig. 4D). With the application of the *directional* method, the rate of misclassification was reduced to 0.9%, and strikingly, all the mES cells were correctly classified. These results indicate that application of the *directional* method improves the quantification estimates and can improve classification by hierarchical clustering.

## Discussion

UMIs can be utilized across a broad range of sequencing techniques; however, bioinformatic methods to leverage the information from UMIs have yet to be standardized. In particular, others have noted the problem of UMI errors, but the solutions applied are varied (Islam et al. 2014; Bose et al. 2015). The *adjacency* and *directional* methods we set out here are, to our knowledge, novel approaches to remove PCR duplicates when using UMIs. By comparing these methods to previous methods with simulated data, we observed that our methods are superior at estimating the true number of unique molecules. Of the three network-based methods, *directional* was the most robust over the simulation conditions and should be preferred. We note that the performance of all network-based methods will decrease as the number of aligned reads at a genomic locus approaches the number of possible UMIs; however, this is an intrinsic issue with UMIs and not one that can be solved computationally post-sequencing. For this reason, we recommend all experiments to use UMIs of at least 8 bp in length and to use longer UMIs for higher sequencing depth experiments. The simulations also indicated that very long UMIs actually decrease the accuracy of quantification when not accounting for UMI errors, since the UMIs are more likely to accumulate errors. For experiments utilizing long UMIs, network-based methods therefore show an even greater performance relative to the *unique* method. The simulations provide an insight into the impact on quantification accuracy and indicate that application of an error-aware method is even more important with higher sequencing depth. This is perhaps most pertinent for single-cell RNA-seq, as cost decreases continue to drive higher sequencing depths.

The analysis of iCLIP and single-cell RNA-seq and data sets established that UMI errors are present in all of the data sets tested and that quantification accuracy could therefore be improved by modeling these errors during the de-duplication step. The frequency of UMI indels was far less than UMI errors, suggesting only minimal gains would be achieved by also considering UMI indels. We observed an improved distribution of edit distances for all samples when using network-based methods to detect PCR duplicates, although theoretical reasoning and empirical evidence suggests that the extent of the errors depends on the quality of the sequencing base calls and the sequencing depth, as confirmed by the simulations.

Modeling UMI errors yielded improvements in single-cell RNA-seq sample clustering, demonstrating the value of considering UMI errors. Because iCLIP aims to identify specific bases bound by RNA binding proteins, data sets have a high level of PCR duplication. The effects of UMI errors are therefore particularly strong, creating the impression of reproducible cross-linking sites within a replicate but not between replicates; for example, only 21% of positions with two or more tags in SRSF7 replicate 1 had any tags in replicates 2 or 3 when naïve de-duplication was used, but this increased to 59% when the *directional* method was used (Fig. 3B). Application of the network-based methods increases the correlation between replicates in all cases, with larger differences in samples in which PCR duplication was higher. From the results of the simulation and real data analyses, we recommend the use of an error-aware method to identify PCR duplicates whenever UMIs are used.

We provide our methods within the open-source UMI-tools software (https://GitHub.com/CGATOxford/UMI-tools) (Supplemental File 1), which can easily be integrated into existing pipelines for analysis of sequencing techniques utilizing UMIs.

## Methods

### Simulation

To simulate the effects of errors on UMI counts, an initial number of UMIs were generated at random, with a uniform random probability of amplification [0.8–1.0] assigned to each initial UMI. To simulate a PCR cycle, each UMI was selected in turn and duplicated according to its probability of amplification. Polymerase errors were also added randomly at this stage, and any resulting new UMI sequences assigned new probabilities of amplification. Following multiple PCR cycles, a defined number of UMIs were randomly sampled to model the sampling of reads during sequencing (“sequencing depth”), and sequencing errors were introduced at a given probability, with all errors (e.g., A → T, C → G) being equally likely. The number of true UMIs within the sampled UMIs was then estimated from the final pool of UMIs using each method. To test the performance of the methods under a variety of simulation parameters, each parameter was varied in turn. The following values are the range of the parameter values tested with the value used for all other simulations in parentheses: sequencing depth 10–400 (100), number of initial UMIs 10–100 (20), UMI length 6–16 (8), DNA polymerase error rate 1 × 10^−3^–1 × 10^−7^ (1 × 10^−5^), sequencing error rate 1 × 10^−1^–1 × 10^−5^ (1 × 10^−3^), number of PCR cycles 4–12 (6), and minimum amplification probability 0.1–1 (0.8). The maximum amplification probability was set at 1, with the probability of amplification for each UMI drawn from a uniform distribution.

### Real data

Re-analysis of the iCLIP and single-cell RNA-seq data was performed with in-house pipelines following the methods described in the original publication with exceptions as highlighted below. Pipelines are available at https://GitHub.com/CGATOxford/UMI-tools_pipelines and as Supplemental File 2.

#### iCLIP

Raw sequence was obtained from the European Nucleotide Archive (accessions SRP059277 and ERR039854) (Tollervey et al. 2011; Müller-McNicoll et al. 2016). Raw sequences were processed to move the UMI sequences to the read name using “umi_tools extract”. Sample barcodes were verified and removed, and the adapter sequence was removed from the 3′ end of reads using the reaper tool from the Kraken package (version 15-065) (Davis et al. 2013) with parameters “-3p-head-to-tail 2 -3p-prefix 6/2/1”. Reads were mapped to the same genome as the original publication (mm9 for SRSF data set, hg19 for the TDP43 data set) using Bowtie version v1.1.2 (Langmead et al. 2009) with the same parameters as the original publications (-v 2 -m 10 -a).

We measured the rate at which UMIs might represent indel mutations by noting that an indel in the UMI sequence would cause the final base of the presumed UMI to match the genomic base at position −1 relative to the mapping location of the read. Thus, we examined each UMI at a particular position and tested for the presence of a UMI that would correspond to a 1-bp deletion existing at the following base. We compared this to the situation when the UMIs at the following base were randomized, respecting the number of UMIs at the position and the genome-wide usage of each UMI. Enrichment was defined as the count at the unrandomized positions compared to the count at the randomized positions. We calculated this metric for one replicate of each pull down from SRP059277. See the *Examining_indels* notebook in the *UMI-tools_pipelines* repository (Supplemental File 2).

Mapped reads were de-duplicated using “umi_tools dedup” using each of the possible methods and edit distance distribution produced using the “--output-stats” option. For the *cluster* method, only the “--further-stats” option was used to output statistics on the distribution of network topology types.

Significant bases were produced by comparing tag count height at each position compared to randomized profiles (König et al. 2010), and bases with FDR < 0.05 retained.

Coverage over exons was calculated by collapsing Ensembl 67 transcripts. Where exons overlapped, they were restricted to their intersection, and the number of reads mapped to significant bases were counted for each exon. Exons that contained no tags in any sample were removed (König et al. 2010). Spearman's ρ between all pairwise combinations of replicates of pull downs for the same protein were calculated and averaged for each replicate.

Reproducibility between replicates was calculated as per König et al. (2010). Bases with a depth greater than two were identified in the sample in question, and then the fraction of these bases that had one or more tags in other replicates was calculated.

#### Single-cell RNA-seq

For both data sets, raw data was downloaded from Gene Expression Omnibus (http://www.ncbi.nlm.nih.gov/geo). For the SCRB-seq data (GSE53638) (Soumillon et al. 2014), a single Day 0 (SRR1058003) and Day 14 (SRR1058023) sample were obtained. For the inDrop data (GSE65525) (Klein et al. 2015), the mouse ES cells sample 1 (SRR1784310), mouse ES cells LIF-, 2 d (SRR1784313), mouse ES cells LIF-, 4 d (SRR1784314), and mouse ES cells LIF-, 7 d (SRR1784315) samples were obtained. FASTQ files were extracted using the SRA toolkit. The sequence read filtering, preparation, and alignment differed for the two data sets. In both cases, one of the paired end reads contained adapter barcodes and UMI, and the other read pair contained sequence for alignment. In addition, with the inDrop data, the position of the UMI within the read varied depending on the length of the cell barcode. For this reason, for both data sets, the UMIs had to be extracted from the reads with bespoke code rather than using UMI-tools *extract*.

For SCRB-seq samples, the UMI was extracted from read 2 and appended onto the read identifier of read 1 to generate a single-end FASTQ. Reads were filtered out if any of the following conditions were not met: Phred sequence quality of all cell barcode bases ≥10 and all UMI bases ≥30 and cell barcode matched expected cell barcodes. A reference transcriptome was built comprising all human protein-coding genes (Ensembl v75, hg19) and the ERCC spike-ins. Because expression quantification was being performed at the gene level, overlapping transcripts from the same gene were merged so that each gene contained a single transcript covering all exons from all transcripts. Reads were aligned to the reference transcriptome using BWA Aln (Li and Durbin 2009) with the following parameters: “*-l 24 –k 2*” to set seed length to 24 bp, and mismatches allowed in the seed to two.

For inDrop samples, the cell barcode and UMI were extracted from read 1, and read 2 was written out to a single-end FASTQ file with the cell barcode incorporated into the file name and the UMI appended to the read identifier. Only reads containing the adapter sequence (allowing two mismatches) were retained. For each sample, only reads containing one of the *n* most abundant cell barcodes were retained, where *n* was the number of cells in a given sample. The resulting single-end reads were filtered using Trimmomatic v0.32 (Bolger et al. 2014) with the following options—“*LEADING:28 SLIDINGWINDOW:4:20 MINLEN:19*”—to remove bases with Phred quality scores below 28 from the 5′ end, scan the reads in 4-bp sliding windows and trim when average quality score falls below 20, and retain all reads at least 19 bp in length following trimming. Our alignment procedure is a deviation from the method used by Klein et al. (2015), which involved alignment of reads to a reference transcriptome containing all transcripts (e.g., not collapsed into one gene model), reporting up to 200 alignments per read, and dealing with multimapping alignments in a downstream step. As this method was not compatible with our de-duplication method, we took a simpler approach. A reference transcriptome was built comprising all mouse protein-coding genes (Ensembl v78, mm10) plus ERCC spike-ins. Because expression quantification was being performed at the gene level, overlapping transcripts from the same gene were merged so that each gene contained a single transcript covering all exons from all transcripts. Reads were aligned to the reference transcriptome with Bowtie v1.1.2 (Langmead et al. 2009) with the following options—“*-n1 -l 15 -M 1 --best --strata*”—to allow one mismatch, set seed length to 15 bp, and report only one alignment where multiple “best” alignments were found. The seed length and mismatch parameters were the same as the Klein et al. (2015) alignment method.

Following alignment, de-duplication was performed with UMI-tools dedup with *unique*, *percentile*, and *directional* used in turn. Both data sets were generated with sequencing methods that generate reads with different alignment coordinates from the same initial DNA fragment (SCRB-seq, CEL-Seq). De-duplication was therefore performed with the “*--per-contig*” option so that the UMI and the contig (in this case, gene) rather than the exact alignment coordinates were used to identify duplicate reads. The “--stats-output” and “--further-stats” options were used to generate summary statistics for the alignment files before and after de-duplication. Gene expression was quantified by counting the number of remaining reads per gene following de-duplication.

#### Exploratory gene expression analysis

PCA was performed in R (R Core Team 2015) using the *prcomp* function*.* Hierarchical clustering was performed in R using the *hclust* function, and heatmaps were generated using the *heatmap.2* function from the gplots package. Clustering was performed using the distance measure (1 − Spearman's correlation coefficient), and clustering method “ward.D2”. Because many genes show very low expression in the SCRB-seq data, the top 100 most highly expressed genes were selected for clustering.

### Software availability

UMI-tools is available from PyPI (package: umi_tools) and conda (channel: https://conda.anaconda.org/toms; package: umi_tools) or GitHub (https://GitHub.com/CGATOxford/UMI-tools). Analyses conducted in this manuscript used version 0.2.6 (archived on Zenodo as https://doi.org/10.5281/Zenodo.165403 and in Supplemental File 1). Analyses were performed using automated Python pipelines. iCLIP specific analyses were completed using the iCLIPlib Python library (Supplemental File 2; I Sudbery, in prep.). Figures were created by Python pipelines or in Jupyter notebooks using the ggplot2 package (Wickham 2009) unless otherwise noted. All pipelines, notebooks, and other code, along with configuration files used, are available from the GitHub repository (https://GitHub.com/CGATOxford/UMI-tools_pipelines), archived on Zenodo (https://doi.org/10.5281/zenodo.215974), and in Supplemental File 2.

## Supplementary Material

Supplemental Material

## References

[SMITHGR209601C1] Aird D, Ross MG, Chen WS, Danielsson M, Fennell T, Russ C, Jaffe DB, Nusbaum C, Gnirke A. 2011 Analyzing and minimizing PCR amplification bias in Illumina sequencing libraries. Genome Biol 12: R18. 21338519 10.1186/gb-2011-12-2-r18 PMC3188800

[SMITHGR209601C2] Bolger AM, Lohse M, Usadel B. 2014 Trimmomatic: a flexible trimmer for Illumina sequence data. Bioinformatics 30: 2114–2120. 24695404 10.1093/bioinformatics/btu170 PMC4103590

[SMITHGR209601C3] Bose S, Wan Z, Carr A, Rizvi AH, Vieira G, Pe'er D, Sims PA. 2015 Scalable microfluidics for single cell RNA printing and sequencing. Genome Biol 16: 120. 26047807 10.1186/s13059-015-0684-3 PMC4487847

[SMITHGR209601C4] Collins JE, Wali N, Sealy IM, Morris JA, White RJ, Leonard SR, Jackson DK, Jones MC, Smerdon NC, Zamora J, 2015 High-throughput and quantitative genome-wide messenger RNA sequencing for molecular phenotyping. BMC Genomics 16: 578. 26238335 10.1186/s12864-015-1788-6 PMC4524448

[SMITHGR209601C5] Davis MPA, van Dongen S, Abreu-Goodger C, Bartonicek N, Enright AJ. 2013 Kraken: a set of tools for quality control and analysis of high-throughput sequence data. Methods 63: 41–49. 23816787 10.1016/j.ymeth.2013.06.027 PMC3991327

[SMITHGR209601C6] He Q, Johnston J, Zeitlinger J. 2015 ChIP-nexus enables improved detection of *in vivo* transcription factor binding footprints. Nat Biotechnol 33: 395–401. 25751057 10.1038/nbt.3121 PMC4390430

[SMITHGR209601C7] Hug H, Schuler R. 2003 Measurement of the number of molecules of a single mRNA species in a complex mRNA preparation. J Theor Biol 221: 615–624. 12713944 10.1006/jtbi.2003.3211

[SMITHGR209601C8] Islam S, Zeisel A, Joost S, La Manno G, Zajac P, Kasper M, Lönnerberg P, Linnarsson S. 2014 Quantitative single-cell RNA-seq with unique molecular identifiers. Nat Methods 11: 163–166. 24363023 10.1038/nmeth.2772

[SMITHGR209601C9] Karlsson K, Sahlin E, Iwarsson E, Westgren M, Nordenskjöld M, Linnarsson S. 2015 Amplification-free sequencing of cell-free DNA for prenatal non-invasive diagnosis of chromosomal aberrations. Genomics 105: 150–158. 25543032 10.1016/j.ygeno.2014.12.005

[SMITHGR209601C10] Kivioja T, Vähärautio A, Karlsson K, Bonke M, Enge M, Linnarsson S, Taipale J. 2012 Counting absolute numbers of molecules using unique molecular identifiers. Nat Methods 9: 72–74. 10.1038/nmeth.1778 22101854

[SMITHGR209601C11] Klein AM, Mazutis L, Akartuna I, Tallapragada N, Veres A, Li V, Peshkin L, Weitz DA, Kirschner MW. 2015 Droplet barcoding for single-cell transcriptomics applied to embryonic stem cells. Cell 161: 1187–1201. 26000487 10.1016/j.cell.2015.04.044 PMC4441768

[SMITHGR209601C12] König J, Zarnack K, Rot G, Curk T, Kayikci M, Zupan B, Turner DJ, Luscombe NM, Ule J. 2010 iCLIP reveals the function of hnRNP particles in splicing at individual nucleotide resolution. Nat Struct Mol Biol 17: 909–915. 20601959 10.1038/nsmb.1838 PMC3000544

[SMITHGR209601C14] Langmead B, Trapnell C, Pop M, Salzberg SL. 2009 Ultrafast and memory-efficient alignment of short DNA sequences to the human genome. Genome Biol 10: R25. 19261174 10.1186/gb-2009-10-3-r25 PMC2690996

[SMITHGR209601C16] Li H, Durbin R. 2009 Fast and accurate short read alignment with Burrows–Wheeler transform. Bioinformatics 25: 1754–1760. 19451168 10.1093/bioinformatics/btp324 PMC2705234

[SMITHGR209601C17] Macosko EZ, Basu A, Satija R, Nemesh J, Shekhar K, Goldman M, Tirosh I, Bialas AR, Kamitaki N, Martersteck EM, 2015 Highly parallel genome-wide expression profiling of individual cells using nanoliter droplets. Cell 161: 1202–1214. 26000488 10.1016/j.cell.2015.05.002 PMC4481139

[SMITHGR209601C18] Marinier E, Brown DG, McConkey BJ. 2015 Pollux: platform independent error correction of single and mixed genomes. BMC Bioinformatics 16: 10. 25592313 10.1186/s12859-014-0435-6 PMC4307147

[SMITHGR209601C19] McCloskey ML, Stöger R, Hansen RS, Laird CD. 2007 Encoding PCR products with batch-stamps and barcodes. Biochem Genet 45: 761–767. 17955361 10.1007/s10528-007-9114-x

[SMITHGR209601C20] Müller-McNicoll M, Botti V, de Jesus Domingues AM, Brandl H, Schwich OD, Steiner MC, Curk T, Poser I, Zarnack K, Neugebauer KM. 2016 SR proteins are NXF1 adaptors that link alternative RNA processing to mRNA export. Genes Dev 30: 553–566. 26944680 10.1101/gad.276477.115 PMC4782049

[SMITHGR209601C21] R Core Team. 2015 R: a language and environment for statistical computing. R Foundation for Statistical Computing, Vienna, Austria http://www.R-project.org/.

[SMITHGR209601C22] Rittié L, Perbal B. 2008 Enzymes used in molecular biology: a useful guide. J Cell Commun Signal 2: 25–45. 18766469 10.1007/s12079-008-0026-2 PMC2570007

[SMITHGR209601C23] Schirmer M, Ijaz UZ, D'Amore R, Hall N, Sloan WT, Quince C. 2015 Insight into biases and sequencing errors for amplicon sequencing with the Illumina MiSeq platform. Nucleic Acids Res 43: e37. 25586220 10.1093/nar/gku1341 PMC4381044

[SMITHGR209601C24] Schirmer M, D'Amore R, Ijaz UZ, Hall N, Quince C. 2016 Illumina error profiles: resolving fine-scale variation in metagenomic sequencing data. BMC Bioinformatics 17: 125. 26968756 10.1186/s12859-016-0976-y PMC4787001

[SMITHGR209601C25] Schloss PD, Gevers D, Westcott SL. 2011 Reducing the effects of PCR amplification and sequencing artifacts on 16s rRNA-based studies. PLoS One 6: e27310. 22194782 10.1371/journal.pone.0027310 PMC3237409

[SMITHGR209601C26] Schmitt MW, Kennedy SR, Salk JJ, Fox EJ, Hiatt JB, Loeb LA. 2012 Detection of ultra-rare mutations by next-generation sequencing. Proc Natl Acad Sci 109: 14508–14513. 22853953 10.1073/pnas.1208715109 PMC3437896

[SMITHGR209601C27] Shiroguchi K, Jia TZ, Sims PA, Xie XS. 2012 Digital RNA sequencing minimizes sequence-dependent bias and amplification noise with optimized single-molecule barcodes. Proc Natl Acad Sci 109: 1347–1352. 22232676 10.1073/pnas.1118018109 PMC3268301

[SMITHGR209601C28] Sims D, Sudbery I, Ilott NE, Heger A, Ponting CP. 2014 Sequencing depth and coverage: key considerations in genomic analyses. Nat Rev Genet 15: 121–132. 24434847 10.1038/nrg3642

[SMITHGR209601C29] Soumillon M, Cacchiarelli D, Semrau S, van Oudenaarden A, Mikkelsen TS. 2014 Characterization of directed differentiation by high-throughput single-cell RNA-Seq. bioRxiv 10.1101/003236.

[SMITHGR209601C30] Tollervey JR, Curk T, Rogelj B, Briese M, Cereda M, Kayikci M, Hortobágyi T, Nishimura AL, Župunski V, Patani R, 2011 Characterising the RNA targets and position-dependent splicing regulation by TDP-43; implications for neurodegenerative diseases. Nat Neurosci 14: 452–458. 21358640 10.1038/nn.2778 PMC3108889

[SMITHGR209601C31] Vollmers C, Sit RV, Weinstein JA, Dekker CL, Quake SR. 2013 Genetic measurement of memory B-cell recall using antibody repertoire sequencing. Proc Natl Acad Sci 110: 13463–13468. 23898164 10.1073/pnas.1312146110 PMC3746854

[SMITHGR209601C32] Waugh C, Cromer D, Grimm A, Chopra A, Mallal S, Davenport M, Mak J. 2015 A general method to eliminate laboratory induced recombinants during massive, parallel sequencing of cDNA library. Virol J 12: 55. 25879746 10.1186/s12985-015-0280-x PMC4403950

[SMITHGR209601C33] Whalen S, Truty RM, Pollard KS. 2016 Enhancer–promoter interactions are encoded by complex genomic signatures on looping chromatin. Nat Genet 48: 488–496. 27064255 10.1038/ng.3539 PMC4910881

[SMITHGR209601C34] Wickham H. 2009 ggplot2: elegant graphics for data analysis. Springer-Verlag, New York.

[SMITHGR209601C35] Yaari G, Kleinstein SH. 2015 Practical guidelines for B-cell receptor repertoire sequencing analysis. Genome Med 7: 121. 26589402 10.1186/s13073-015-0243-2 PMC4654805

