# Supplementary material for: UMI-tools: modeling sequencing errors in Unique Molecular Identifiers to improve quantification accuracy
Source: Genome Res. 2017 Mar;27(3):491–9. doi: 10.1101/gr.209601.116 (PMC5340976; doi:10.1101/gr.209601.116)
Supplement: Supplemental Material [file supp_27_3_491__index.html]

UMI-tools: modeling sequencing errors in Unique Molecular Identifiers to improve quantification accuracy — Supplemental Material 

# UMI-tools: modeling sequencing errors in Unique Molecular Identifiers to improve quantification accuracy

## Supplemental Material

- Supplementary\_Figures.pdf
- Supplementary\_File1.zip
- Supplementary\_File2.zip
