# Supplementary material for: UMI-tools: modeling sequencing errors in Unique Molecular Identifiers to improve quantification accuracy
Source: Genome Res. 2017 Mar;27(3):491–9. doi: 10.1101/gr.209601.116 (PMC5340976; doi:10.1101/gr.209601.116)
Supplement: Supplemental Material [file supp_gr.209601.116_Supplementary_Figures.pdf]

Supplementary Figures

A

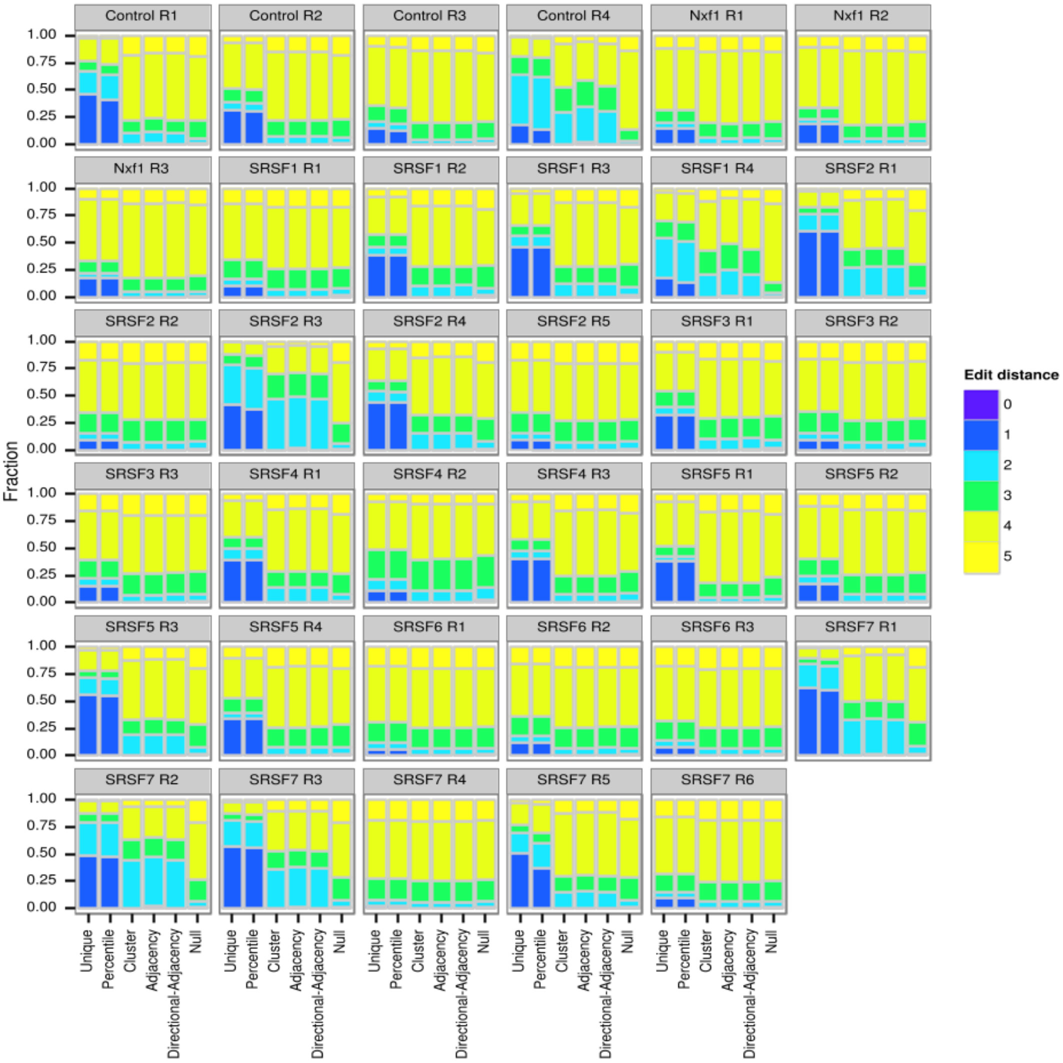

B

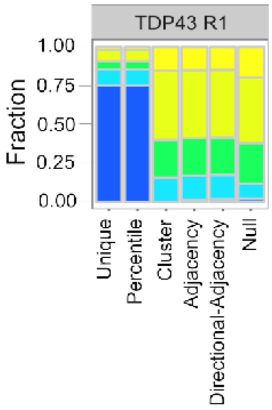

Supplementary Figure 1. Edit distances for all iCLIP replicates

Average edit distances between UMIs with the same alignment coordinates. Genomic positions with a single UMI are not shown. Null = Null expectation from random sampling of UMIs. **A** Further replicates from the SRSF dataset showing that effects vary between replicates, but are not specific the replicate 1. **B** Results from a TDP43 iCLIP dataset showing effects are not limited to a single experiment (Tollervey et al., 2011)

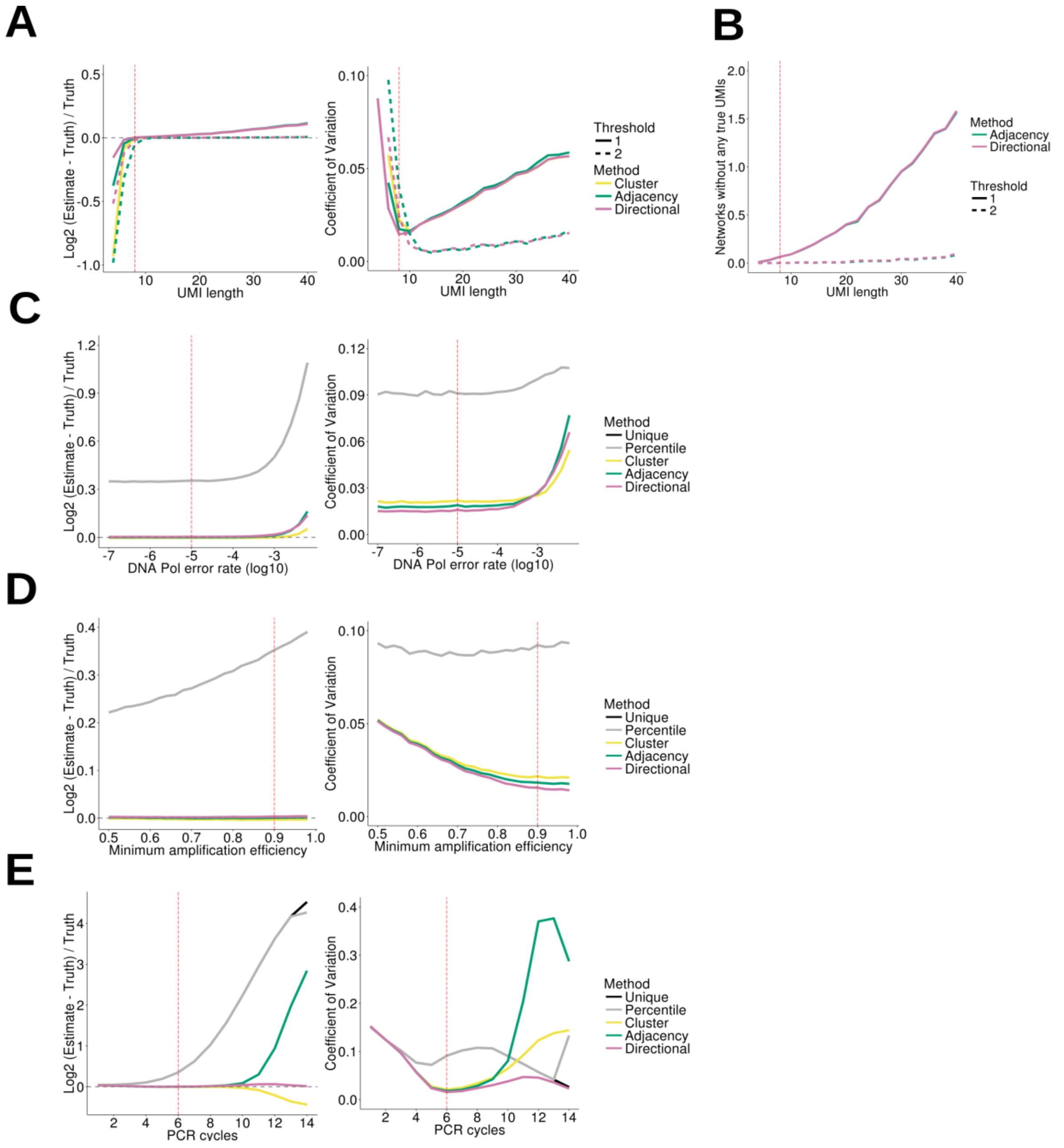

Supplementary Figure 2. Comparison of methods with simulated data

A, C-E. All simulation parameters one are held constant, with the remaining parameter varied as shown on the x-axis. Left plot shows the accuracy of quantification, presented as the log2-transformed normalised difference between the estimate and ground truth. Ground truth = 20 UMIs. The dashed red line represents the value used for this parameter in all other simulations. The dashed grey line represents perfect accuracy. Right plot shows the coefficient of Variation (standard deviation / mean). **A**.Accuracy of network-based methods with very long UMIs. Unique and

percentile are not shown. Adjacency and directional methods were run with the edit distance threshold for edges between nodes set at 1 or 2. **B.**The effect of UMI length on the number of networks formed without a single true UMI. These networks are formed when two errors occur in a single UMI and the intermediate UMI is not observed. Only adjacency and directional methods are shown here since the cluster and adjacency method form the exact same networks.**E.** The sequencing depth has been increased to 50% to demonstrate the slightly increased accuracy for percentile when sequencing depth and number of PCR cycles are both high

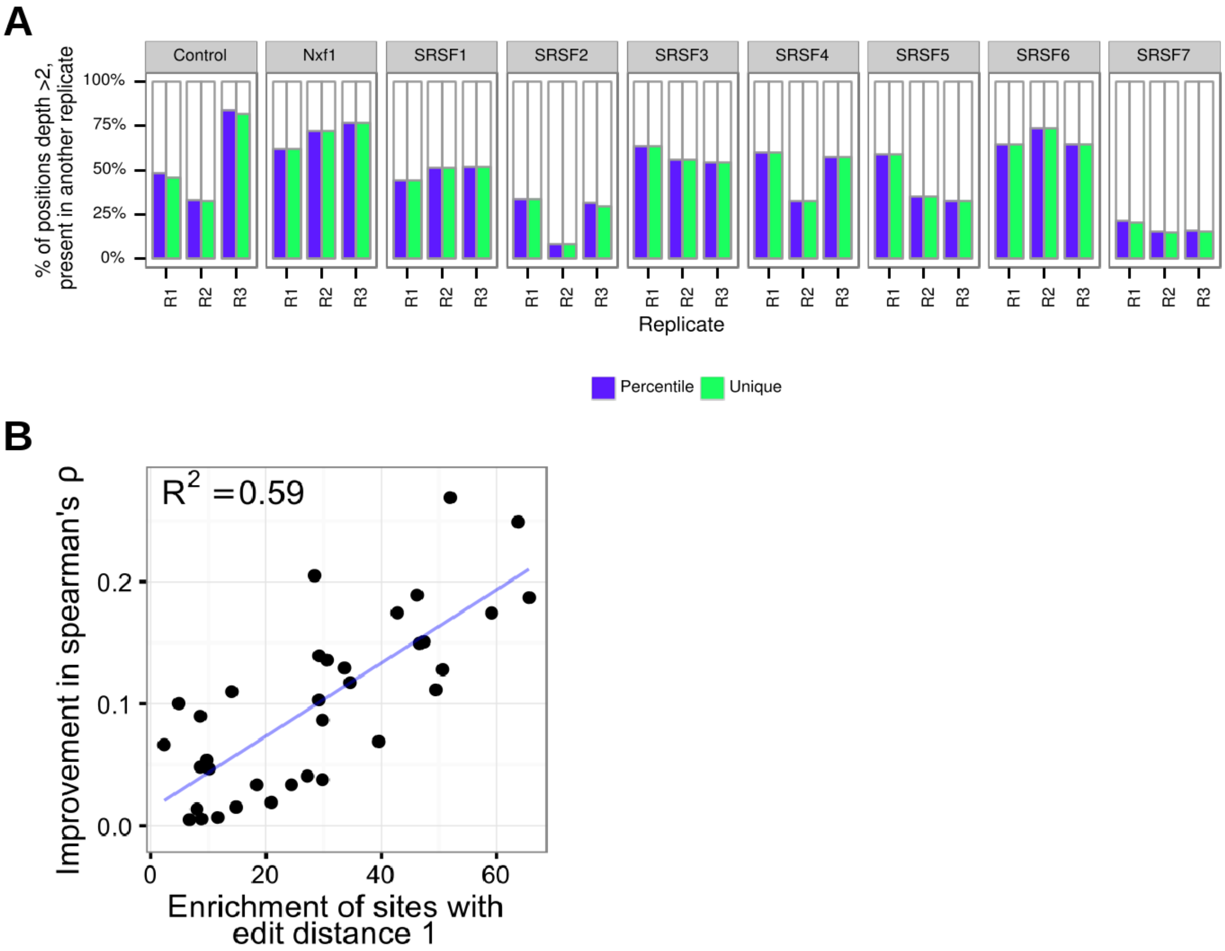

Supplementary Figure 3. UMI-Tools improves reproducibility between iCLIP replicates

A. iCLIP reproducibility as represented by the percentage of positions with >2 tags also cross-linked in at least one of 2 other replicates. No improvement is observed with **B.** Correlation between enrichment for sites with an average edit distance of 1 following unique de-deduplication and the improvement in Spearman's ρ following directional adjacency de-duplication.
