# Supplementary material for: UMI-tools: modeling sequencing errors in Unique Molecular Identifiers to improve quantification accuracy
Source: Genome Res. 2017 Mar;27(3):491–9. doi: 10.1101/gr.209601.116 (PMC5340976; doi:10.1101/gr.209601.116)
Supplement: Supplemental Material [file supp_gr.209601.116_Supplementary_File2.zip › UMI-tools_pipelines-0.0.4/iCLIPlib/pipeline_iCLIP/pipeline_docs/themes/cgat/layout.html]

{#
default/layout.html
~~~~~~~~~~~~~~~~~~~
Sphinx layout template for the default theme.
:copyright: Copyright 2007-2010 by the Sphinx team, see AUTHORS.
:license: BSD, see LICENSE for details.
#}
{% extends "basic/layout.html" %}
{% set script\_files = script\_files + ['\_static/sorttable.js'] %}
