# Supplementary figures and images for: UMI-tools: modeling sequencing errors in Unique Molecular Identifiers to improve quantification accuracy
Source: Genome Res. 2017 Mar;27(3):491–9. doi: 10.1101/gr.209601.116 (PMC5340976; doi:10.1101/gr.209601.116)

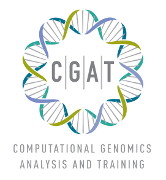

Supplement: Supplemental Material [file supp_gr.209601.116_Supplementary_File2.zip › UMI-tools_pipelines-0.0.4/iCLIPlib/pipeline_iCLIP/pipeline_docs/_templates/cgat_logo.png]
